# Retrieval of the Extreme Values under Deadline Constraints in Wireless Sensor Networks

**DOI:** 10.3390/s110505229

**Published:** 2011-05-12

**Authors:** Seung Jun Baek, Xiaohan Yu, Kyogu Lee, Hyunhak Kim

**Affiliations:** 1 Department of Computer and Communications, Korea University, Anam-dong Seongbuk-gu, 130-701, Seoul, Korea; E-Mail: yuxiaohan188@126.com; 2 Department of Digital Contents Convergence, Seoul National University, Seoul, Korea; E-Mail: kglee@snu.ac.kr; 3 Electronics and Telecommunications Research Institute, 138 Gajeongno, Yuseong-gu, Daejeon, 305-700, Korea; E-Mail: hh.kim@etri.re.kr

**Keywords:** wireless sensor networks, medium access control, distributed algorithm, latency

## Abstract

We consider a problem of retrieving the extreme value among sensed data under deadline constraints in wireless sensor networks with potential applications to alarm systems. The sensed data is mapped to a score which we adopt as a unified measure of the relative urgency of the data. The objective is to retrieve the data with the maximum score. We propose fully distributed schemes for contention based medium access and data combining. The proposed medium access scheme uses a randomized back-off which is controlled based on the score of the data to be transmitted. Data combining techniques are proposed to further suppress unnecessary traffic and reduce contention. The key observation is that one should aggressively prioritize packets with high score, up to an extent that does not incur excessive contention in channel access. Designed to capture such aspect, the proposed scheme is shown to substantially decrease the latency of the retrieval.

## 1. Introduction

Wireless sensor networks (WSN) are networks of battery-operated sensors which are capable of wireless communication. WSNs envision a wide range of applications in environmental and habitat monitoring, military surveillance, building and facility monitoring, and emergency medical care. An important property of WSNs is that they are application-centric, *i.e.*, WSNs are deployed to carry out a specific mission. Thus it is desirable that the design of WSN is adapted to specific tasks so as to optimize the performance in mission execution.

Commonly an objective of WSNs is to provide a *summary* of the sensed data generated in the network. The relevant summary is defined as certain function applied to the raw sensor data [1]. Given the sensor readings *x*_1_, *x*_2_, . . ., *x_n_*, the goal is to compute *f*(*x*_1_, *x*_2_, . . ., *x_n_*). Examples of *f*(·) are mean, max, min, *etc.* When mean function is used, $f(x_{1},\;\ldots ,\;x_{n})\;=\;\sum_{i=1}^{n}x_{i}/n$. In this paper we study the design of WSN with an objective of *extreme* data retrieval. Specifically *f*(·) of interest is max or min, however these two objectives are identical in nature, thus without loss of generality we will focus on max. Note WSNs for such extreme data retrieval is suitable for ‘alarm’ systems [1]. For example suppose *f*(*x*_1_, . . ., *x_n_*) = max*_i_*_=1,...,_*_n_* [*x_i_*] where *x_i_* are the temperature values of the monitoring region. Then the mission can be to identify the highest temperature value and its location so that an alarm is triggered if the value is above a threshold. A crucial aspect of emergency applications is to retrieve the information in a timely manner. We assume that there is a strict deadline for data retrieval, and we focus on maximizing the probability of the retrieval of the extreme data at the deadline.

When the data samples *x*_1_, *x*_2_, . . ., *x_n_* are drawn from random variables (RV) *X*_1_, *X*_2_, . . ., *X_n_* with *different* distributions, it is meaningless to directly compare the values *x*_1_, . . ., *x_n_* or to compute max among the data. To deal with such heterogeneity we will introduce a *score* system which provide a unified measures of the ‘urgency’ of a data sample. Specifically a score represents how much the associated data sample is *deviated* from its usual distribution. Thus the objective is in fact to retrieve the data with the maximum *score*, *i.e.*, the data sample that exhibits the highest degree of ‘abnormality’. The details are explained in Section 3.

We consider a network such that data is collected in a multi-hop manner through a tree topology. The sink is located at the root of the tree. The goal for the sink is to retrieve the extreme data through the tree, and the data gathering tree is assumed to be given. The data collection is initiated at an instant which is not known beforehand. Every sensor begins forwarding a data sample to the sink at the instant. Latency is defined to be the time between the initiating instant and the retrieval of the extreme value among data samples at the sink. We want the latency to meet the deadline requirement. Since the sink only needs to compute the extreme value, there can be substantial redundancy in the traffic generated by the network. If every sensor were to forward every generated reading to the sink, it would incur significant latency and energy consumption. As will be detailed later, a certain class of *f*(·) allow the data to be combined in the intermediate nodes along the route. For such class of *f*(·), max being in the class, intermediate combining of data do not alter the final *f*(·) computed at the sink. A collective term for the intermediate aggregation and combining techniques is *in-network aggregation* [2–4]. A survey on in-network aggregation techniques is provided in [5].

We propose a scheme for latency reduction based on priority-based medium access and data combining. The scheme consists of the following three schemes. The first scheme focuses on the medium access aspects. In our model the sensors contend for channel access in a fully distributed manner. Multiple requests for a single time resource result in a collision. Since the network traffic needs to be aggregated to the sink in a short time, the traffic is bursty in time and space. Thus it is crucial to resolve contention in an efficient way. Intuitively it is preferable to give high priority of channel access to sensors having ‘large’ data values, since we wish the maximum data to arrive at the sink as early as possible. We propose a scheme to implicitly assign such priority by means of randomized back-off which is controlled based on how ‘large’ the data value is. We observe that there exists a trade-off between priority assignment and channel contention. If the priority assignment is highly biased towards a certain class of sensors, it may restrict resource for other classes of sensors. In situations where a number of sensors with restricted resource have to compete, a collision can occur with high probability. Conversely if we treat all the nodes equally, the number of collisions can be reduced, however there will be no gains in latency reduction from prioritizing the nodes with large data values. A contention resolution scheme capturing this aspect is proposed in Section 4.

The second scheme is called *selective forwarding*, and third scheme is to exploit *overhearing* other transmission. These schemes attempt to suppress unnecessary traffic by leveraging the property of the objective max function as follows. Suppose a node receives a packet to be forwarded to the next hop. If any of the previous packets this node has forwarded/received contain a data value *greater* than that of the current packet, the packet is of no use to the sink. Thus this forwarding request can be ignored, which reduces traffic. The overhearing technique exploits the broadcast property of wireless transmission, and the similar principle to that of selective forwarding is applied in order to further decrease traffic.

When the three schemes are combined, a synergistic effect in latency reduction is observed. Specifically the second and third techniques help reducing unnecessary traffic, thus lowering the level of contention and the number of collisions. Due to the aforementioned trade-off between priority assignment and collision, reduced contention enables more *aggressive* assignment of high priority to high data sensors by the first scheme, which further reduces the latency defined above. The main idea is elaborated in Section 4, and is supported by simulation results in Section 5.

## 2. Related Work

Fundamental limits on computing a class of functions called *divisible* functions *f*(·) are studied in [1]. The divisible functions are a class of functions that can be computed in divide-and-conquer manner without affecting the final result. Suppose there are a set of *n* data samples. Divide the set into two arbitrary groups of *k* and *n – k* samples. Let us denote the data of the first and second group by *x*_1_, *x*_2_, . . ., *x_k_* and *x_k_*_+1_, . . ., *x_n_* respectively. If *f*(·) is divisible, *f* (*f*(*x*_1_, . . ., *x_k_*)*, f*(*x_k_*_+1_, . . ., *x_n_*)) = *f*(*x*_1_, *x*_2_, . . ., *x_n_*) holds for any *n* and *k*. The divisible functions enable efficient in-network aggregation. Examples of divisible functions are sum, max and min. Moreover [1] showed that when computing max or min functions, one can achieve particularly low overhead in communications which scales as at most *O*(log(*n*)). This suggests that there is much room for performance improvement in retrieving the extreme data.

A number of data gathering problems aiming at achieving low latency have been studied. The trade-off between energy and data gathering latency is investigated in [6]. The authors study an aggregation strategy for minimizing energy dissipation exploiting the fact that longer transmission time of a packet can save energy expenditure, *i.e.*, a link-level trade-off studied in [7]. A series of works [8–10] have focused on scheduling of data aggregation in order to minimize latency. In [10] a scheduling algorithm of the links in aggregation tree using maximal independent set to achieve a latency bound which scales additively in network radius and maximum degree.

The efficiency of in-network aggregation is characterized by the following trade-off between traffic reduction and latency. A node may wait for sufficient amount of data to be aggregated before forwarding the aggregated information to the next-hop node. The longer a node waits for aggregation, the more the traffic is decreased. The decrease in traffic reduces contention for channel access, which improves the efficiency of in-network aggregation. On the other hand, such waiting time incurs extra latency in the data gathering process. Thus the question is how long nodes should wait before transmitting the aggregated information when there is a deadline constraint. The work of [11] formulates such decision making as semi-Markov Decision Process. They propose an approximation algorithm for finding the optimal waiting time for aggregation in order to maximize the ‘reward’. The reward is defined as a function of the number of samples collected at the sink before deadline. Also [12] proposed a framework based on dynamic programming for maximizing the number of packets reaching the sink under energy and deadline constraints. The work [13] studies a similar problem of maximizing the amount of collected information using dynamic programming. They consider a contention-based MAC using CSMA/CA, and find the optimal waiting time for aggregation and the associated MAC parameters.

Unlike these works we do *not* consider optimizing such waiting times. In the proposed scheme, a node attempts transmission if there is any data to send, and does not wait for further aggregation of data. Instead we focus on distributed, priority-based channel access and techniques specific to retrieving the maximum value. However it is clear that the proposed scheme can be easily extended to schemes such that the nodes wait for the aggregation of data. Such extension should address the problem of optimizing waiting time under the proposed medium access and data combining scheme, which is a possible direction for future study. We also mention that our scheme is fully distributed and has very low overhead that is independent of the network size.

A number of recent works discussed distributed medium access based on priority. Much attention has been paid on designing distributed scheduling algorithms to achieve the maximum throughput of multi-hop wireless networks [14–21]. By assigning channel access priorities based on nodes’ load, e.g., queue length. These works have shown the existence of distributed algorithms for maximum throughput in various network models, e.g., node-exclusive interference and IEEE 802.11-like model with collisions. The details and the relevance to our work will be discussed later in Section 4.4.

In the following we mention additional related works which aim at the reduction of traffic in sensor networks. The works [22,23] have proposed the routing protocols which carry out an efficient transportation of the most excessive values of sensed data. The TiNA scheme [24] is proposed to reduce power consumption by suppressing the generation of new data if its value does not differ from the previously transmitted values by more than certain threshold. A scheme to resolve the traffic congestion in sensor networks so-called funneling effect is proposed in [25] which uses spatially adaptive in-network aggregation. The trade-off between the power savings and preserving the integrity of aggregated information has been investigated in [26].

## 3. Model

**Figure 1.**
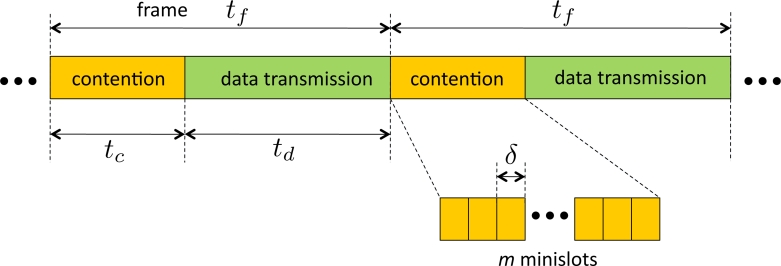
A time diagram for frame structures.

The topology of the network is modeled by a graph *𝒢* = (*V*, *E*) where *V* is set of node indices and *E* is the set of wireless links. We consider a *node exclusive interference* model for the wireless sensors. The primary constraint is that a node cannot simultaneously receive and transmit data. A slight modification is made to the node exclusive model in this paper. We assume that a node can *overhear* the data transmitted by a one-hop neighbor unless the node is either a transmitter or receiver. In the original model it is assumed that only one node can receive from a transmitter, which is mainly for the ease of modeling, however our modified version better captures the broadcast nature of wireless networks. We consider a time-slotted system. The overall time is broken into *frames* where each frame has a constant duration denoted by *t_f_*. A frame is divided into two phases similar to the model used in [18,20]. The first phase, called *contention phase*, is used for contention-based channel access and contention resolution. A contention phase consists of *m* time slots which are called *minislots* of duration *δ*. The minislots are indexed by {1, 2, . . ., *m*}. The duration of the contention phase is denoted by *t_c_*. The second phase is called *data transmission phase*, and is used for data transmission. The data transmission phase has a fixed duration of *t_d_*, e.g., see Figure 1. Nodes are allowed to contend for channel access and transmit data only at the beginning of each frame. We will assume that fine-grained synchronization schemes, such as [27], are available. Thus the nodes are assumed to be synchronized to the timeslots with negligible errors.

The nodes are allowed to begin transmission at only one of the minislots. If there is only one sensor that transmits the earliest in the contention phase, we say the sensor wins the frame. The winner of the frame will transmit during the frame. The transmission from the winning node is assumed to be detectable by the contending nodes. When the contending nodes senses the channel busy, they stop further attempts for contending and wait for the next frame in which they retry transmission. If more than one nodes attempt to transmit in the same minislot, a collision is declared. We assume that such collision can be sensed by contending nodes, *i.e.*, both collision and successful transmissions are ‘channel busy’ events. When a collision occurs, all the contending nodes cease the attempts to transmit on the current frame, and resume contention on the next frame.

We assume the data gathering is globally initiated, and the initiation signal is synchronized to a frame boundary. At the signaling, the measured data is assumed to be available at every node, thus all the nodes initiate transmissions simultaneously. The initiation signaling is assumed to be instantaneously done through a network-wide broadcast by certain ‘supernode’ e.g., an aircraft transmitting a strong signal that can be received by all the nodes in a sensing field. From the initiation signal a deadline *d*, measured in terms of the number of frames, is imposed on the network. Let us denote the number of frames it takes for the sink to retrieve the maximum data value by a RV *T*. Our goal is to maximize ℙ(*T* ≤ *d*). From the definition of the latency, the objective of ‘maximizing the probability of retrieval subject to deadlines’ is equivalent to ‘maximizing the cumulative distribution function (CDF) of the latency’. In Section 5, using simulations, we will demonstrate that the CDF of the latency of the proposed scheme exceeds that of the baseline scheme.

The set of node indices is denoted by *I* = {1, 2, . . ., *n*}. We assume the reading from sensor node *i* ∈ *I* is a continuous RV *X_i_* which has a smooth cumulative distribution function (CDF) *F*_*X_i_*_ (·) and is independent of other sensors’ data. We begin by providing a precise definition of the ‘extreme’ or ‘maximum’ data value. Note the typical motivation behind retrieving extreme data is to detect *abnormality* of sensor readings. The abnormality can be defined as how much a sensor’s reading deviates from its usual statistics. Specifically for a given reading *x_i_* which is a realization of *X_i_*, we should focus on how *unlikely* the event *x_i_* is to occur with respect to its usual statistics seen by Node *i*. Let us denote the distribution function of *X_i_* by *F*_*X_i_*_ (*x_i_*) = ℙ(*X_i_* ≤ *x_i_*). Suppose we have the readings *x*_1_, . . ., *x_n_*, we would like to find the node i* such that

(1) $$i^{*}\;=\;\underset{i\in I}{\text{argmin}}\;[𝕇(X_{i}\;>\;x_{i})]\;=\;\underset{i\in I}{\text{argmax}}\;[F_{X_{i}}\;(x_{i})]$$

and x_i*_. This approach is particularly useful if the data collected by sensors modeled by RVs with heterogeneous distributions. For example, suppose *X_i_* is a Gaussian RV with mean *μ_i_* and variance $\sigma_{i}^{2}$, or *X_i_* ∼ *N* (*μ_i_*, $\sigma_{i}^{2}$). If *μ_i_* and *σ_i_* were to be identical, for a given readings *x_i_* and *x_j_*, we would be able to directly compare *x_i_* and *x_j_*. In such case the objective reduces to finding i* = argmax*_i_*_∈_*_I_* [*x_i_*], *i.e.*, finding the literally maximum data. By contrast if *μ_i_* and *σ_i_* are heterogeneous among sensors, such direction comparison is not possible—only the comparison through *F*_*X_i_*_ (*x_i_*) and *F*_*X_j_*_ (*x_j_*) would be appropriate for our purpose. It is assumed that every node knows the CDF of its sensed data. Also the sink is aware of *F*_*X_i_*_ (·), ∀*i* ∈ *I*. Let us define *Y_i_* := *F*_*X_i_*_ (*X_i_*) and also denote a realization of the data by *y_i_* := *F*_*X_i_*_ (*x_i_*). We call *Y_i_* the *score* of the data of node *i*. By definition the score *Y_i_* is a uniform RV on [0,1]. Since the data generated in the network is assumed to be independent across sensors, the problem is effectively retrieving max*_i_*_∈_*_I_* [*Y_i_*], or the maximum among *n* i.i.d. uniform RVs. Instead of reporting the actual readings *x_i_*, sensors generate/forward data packets containing *y_i_* and *i*, the node ID. Since the sink is aware of *F*_*X_i_*_ (·), it can recover *x_i_* from *y_i_* if it desires to do so. Thus our objective is to retrieve the data with the maximum *score*.

## 4. Proposed Scheme

The proposed scheme consists of three elements: priority-based medium access, selective forwarding and overhearing.

### 4.1. Priority-Based Medium Access

We propose a fully distributed medium access scheme such that the node with high score is likely to access the channel earlier than those with lower score. To facilitate understanding we begin with the limiting case such that the length of minislots vanish however the contention window remains fixed. Namely we let *δ* = *t_c_/m* and *m* → ∞. Thus choosing a minislot to transmit data corresponds to selecting a real number from the continuous interval [0, *t_c_*] which is the time instant to start transmission. In this case it is easy to find a fully distributed scheme without collision: for example Node *i* sets a timer for channel access at the beginning of each frame where the timeout is set to (1 – *y_i_*)*t_c_*. Then with probability 1 Node i* = argmax_*i*∈*I*_ [*y_i_*] will win the channel access. Thus if Node i* is *k* hops away from the sink, the maximum-score data reaches the sink in exactly *k* frames with probability 1. If we consider a balanced tree for the data gathering tree, the maximum score data is retrieved in *k* ∼ *O*(log(*n*)) frames.

In practice however, the minislot *δ* cannot be indefinitely small. *δ* should account for propagation delay and should be sufficiently long such that the transmission from a station can be detected by its neighboring nodes [28]. For example in 802.11a, *δ* is given by 9 *μs*. We propose a fully distributed priority based scheme when *δ* is strictly positive and *m* is finite. The idea in the above case of continuous contention window with vanishing *δ* was to *defer* transmission based on the score. For *δ* > 0 with finite *m*, however, one should not defer transmission in a deterministic manner, otherwise once multiple nodes collide at a frame they will repeat colliding in all subsequent frames, resulting in a deadlock. In the proposed scheme a node defers the *participation* in channel contention, by remaining idle during the first several minislots, say *k* < *m* minislots. Note *k* is determined based on the score. Since *m* is fixed, this has an effect of *shrinking* the individual contention window to *m* – *k* for the sensor. We refer to such shrunk contention window size as the *effective contention window* (ECW) for the node. The sensor selects a minislot for transmission randomly and uniformly over the remaining *m* – *k* minislots.

Specifically the proposed scheme operates as follows. We introduce global system parameters denoted by *β* which is a strictly positive integer, and *γ* which is a strictly positive real number. Details on these parameters will be explained later. For Sensor *i* let us denote the data to be transmitted/forwarded by the score *y_i_* ∈ [0, 1]. The counter *c* ∈ ℤ_+_ is incremented each time collision occurs, and is reset to 0 once a transmission is successful.

1. Set the window length *w* as
  (2) $$w\;=\;(2^{c}\;\cdot \;\left\lceil m\;\cdot \;{\left(y_{i}\right)}^{\gamma }\right\rceil \;+\;\beta )\wedge m$$
  where *a* ∧ *b* denotes min(*a, b*) and ⌈·⌉ denotes the ceiling function.
2. Select a minislot randomly from *m* – *w* + 1, . . ., *m* with the equal probability of *w*^−1^.

The details are provided as follows. The idea is that a node delays channel contention by the number of minislots that increases with decreasing *y_i_*. Thus the sensors with higher *y_i_* gets higher chance of transmission, since such sensors are likely to participate in contention earlier during the contention phase. The choice of the effective window size *w* given by Equation (2) renders the size be roughly proportional to $y_{i}^{\gamma }$, if we ignore *β*. The term 2*^c^* accounts for binary exponential back-off (BEB), *i.e.*, ECW size increases exponentially with the number of successive collisions.

**Figure 2.**
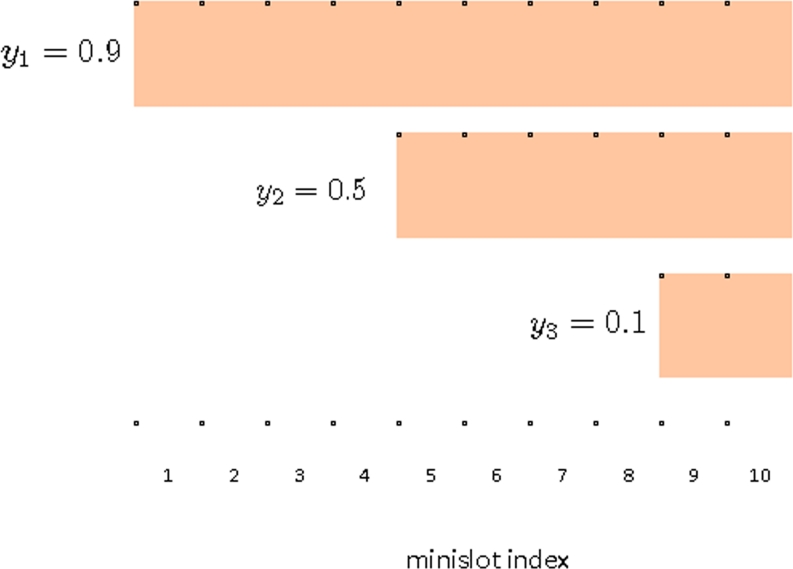
Examples of contention window.

An example is illustrated in Figure 2 assuming *m* = 10, *γ* = 1, *c* = 0 and *ß* = 1. There are three contending sensors with ID 1, 2 and 3. The scores for the sensors are given by *y*_1_ = 0.9, *y*_2_ = 0.5 and *y*_3_ = 0.1. From Equation (2), ECW size for Sensor 1 is *m*, thus it fully utilizes the contention phase period. Sensor 2 has the score 0.5, thus from Equation (2), its ECW size is given by 6. Sensor 2 picks a minislot randomly from minislot index 5, 6, . . ., 10. Thus the probability of selecting a minislot within ECW is 1/6. Sensor 3 has a low score of *y*_3_ = 0.1, thus gets ECW size of 2. The purpose of the parameter *ß* is to set a lower bound on the size of ECW. Since *ß* ≥ 1, the lower bound on *w* is at least 1.

#### System Parameters

**Figure 3.**
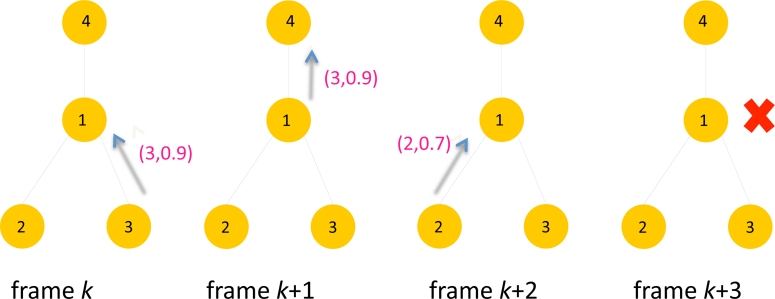
A plot of *y^γ^* for *y* ∈ [0, 1] with warping factors *γ* = 1, 2, 3, $\frac{1}{2}$, $\frac{1}{3}$ and 0.

The parameter *γ* is called *warping factor*. As the name suggests scores are nonlinearly distorted by *γ* in determining the ECW. By controlling *γ* one can achieve varying degrees of *aggressiveness* in giving priority to nodes with high scores. To facilitate understanding the role of *γ*, let us consider an illustrated example in Figure 3. The function *y^γ^* has been plotted for *y* ∈ [0, 1] with varying *γ*. Consider four score values *y*_1_, *y*_2_, *z*_1_ and *z*_2_. The pair of high scores *y*_1_ and *y*_2_ are close to 1, and the pair of low scores *z*_1_ and *z*_2_ are close to 0.

Consider the case *γ* *>* 1. For high score pairs *y*_1_ and *y*_2_, a small difference between *y*_1_ and *y*_2_ will result in a large difference in the ECW size. Also consider a pair of ‘low’ scores *z*_1_ and *z*_2_, the difference between the ECW size is small relative to the difference between *z*_1_ and *z*_2_. Since *w* is monotonically decreasing function in *γ*, the ECW shrinks with increasing *γ* for any fixed score. Thus by increasing *γ* one increases overall chance of collision, however also increases the probability of high score sensors winning the frame.

The case where 0 < *γ* < 1 has the opposite effect. With decreasing *γ*, the sensitivity of the ECW size decreases (resp. increases) for high score (resp. low score) sensors. Furthermore decreasing *γ* has an effect of increasing ECW for all the sensors, thus the probability of collision will decrease. Roughly speaking, small *γ* implies that the sensors are roughly fair in terms of the likelihood of channel access regardless of the scores. In the limiting case where *γ* = 0 all the sensors gets the maximum window size of *m* minislots. We refer to a baseline contention scheme such that every node selects a minislot randomly and uniformly from minislot indices 1, 2, . . ., *m* as UNIFORM scheme. Thus when *γ* = 0, the proposed scheme reduces to UNIFORM scheme.

Thus *γ* quantifies the aggressiveness in prioritizing high score sensors in channel access. Larger *γ* corresponds to higher aggressiveness. The choice of *γ* will be discussed later.

#### Analysis of Transmission Probability

The proposed medium access scheme is analyzed below. Suppose all the *n* users contend for channel access at certain frame. The scores are denoted by *y*_1_, *y*_2_, . . ., *y_n_*. Without loss of generality we assume *y*_1_ ≥ *y*_2_ ≥ . . . ≥ *y_n_*. For Sensor *i*, we would like to find out the probability that Sensor *i* will win the frame. We found that it is complicated to solve for the probability for nonzero *δ*. Instead we will consider an approximation which is a continuous time analogue to the system. We will again assume the ideal case such that *m* → ∞ and *δ* = *t_c_/m* → 0. For further simplicity we will ignore *ß* and assume *γ* = 1 and *c* = 0, and define *t_c_* = 1. Under these assumptions the time instant for transmission, which is analogous to choosing a minislot in the discrete time case, is chosen uniformly over the interval [1 – *y_i_*, 1], ∀*i* ∈ *I*. Thus the size of ECW for Sensor *i* is *y_i_*.

**Proposition 1.** *The probability that Sensor i wins the frame is given by*

(3) $$\sum_{j=0}^{n-i}\left[\left(\prod_{k=1}^{i+j}z_{k}^{-1}\right)\;\left\{\frac{z_{i+j}^{i+j}\;-\;z_{i+j+1}^{i+j}}{i+j}\right\}\right]$$

*where z_j_* := *y_j_*/*y_i_* *for j* = 1, . . ., *n* and *z_n_*_+1_ := 0.

*Proof.* We will assume *i* > 1: it is easy to verify that Equation (3) holds for the case where *i* = 1 using the similar arguments as below. Let us define the time variable *t* such that *t* = 0 at the instant of the beginning of the ECW of Sensor *i*. Consider the probability of Sensor *i* winning the frame during the time interval [0, *y_i_* – *y_i_*_+1_], *i.e.*, Sensor *i* wins the frame prior to the beginning of the ECW of Sensor *i* + 1. Since the probability that Sensor *i* transmits during [*t*, *t* + *dt*] is given by *dt/y_i_*, and in order for this transmission to be successful on Sensors 1, . . ., *i* – 1 must not have transmitted until time *t*. Note the ECW of Sensor *k* for 1 ≤ *k* ≤ *i* – 1 begins at time *y_i_* – *y_k_* which is a negative number. Thus Sensor *k* must not transmit during [*y_i_* – *y_k_*, *t*], and the probability for this event is

$$1\;-\;\frac{y_{k}\;-\;y_{i}\;+\;t}{y_{k}}\;=\;\frac{y_{i}\;-\;t}{y_{k}}$$

Since the above events are mutually independent, the probability of Sensor *i*’s transmission is successful during [0, *y_i_* – *y_i_*_+1_] is given by

(4) $$\int_{0}^{y_{i}-y_{i+1}}\prod_{k=1}^{i-1}\left(\frac{y_{i}\;-\;t}{y_{k}}\right)\;\frac{dt}{y_{i}}$$

By making the change of integration variables *s* = *t/y_i_*, Equation (4) is equal to

(5) $$\left(\prod_{k=1}^{i-1}\frac{y_{i}}{y_{k}}\right)\;\int_{0}^{1-z_{i+1}}{\left(1\;-\;s\right)}^{i-1}ds\;=\;\left(\prod_{k=1}^{i-1}z_{k}^{-1}\right)\;\left\{\frac{1\;-\;z_{i+1}^{i}}{i}\right\}\;=\;\left(\prod_{k=1}^{i}z_{k}^{-1}\right)\;\left\{\frac{z_{i}^{i}\;-\;z_{i+1}^{i}}{i}\right\}$$

where *z_k_*’s follow the above definition. Note the Equation (5) results simply from *z_i_* = 1.

Let us consider the probability that Sensor *i*’s transmission is successful during [*y_i_* – *y_i_*_+1_, *y_i_* – *y_i_*_+2_]. This time interval corresponds to the duration between the beginning of ECW of Sensor *i* + 1 and *i* + 2. Similar to the previous case, when Sensor *i* transmits during [*t, t* + *dt*], Sensor *k* for *k* ∈ {1, . . ., *i* – 1, *i* + 1} must not transmit until time *t*. The probability of such event is

(6) $$\prod_{k=1,k\neq i}^{i+1}\left(\frac{y_{i}\;-\;t}{y_{k}}\right)$$

Thus the probability of Sensor *i* transmission being successful during [*y_i_* – *y_i_*_+1_, *y_i_* – *y_i_*_+2_] is

(7) $$\int_{y_{i}-y_{i+1}}^{y_{i}-y_{i+2}}\prod_{k=1,k\neq i}^{i+1}\left(\frac{y_{i}\;-\;t}{y_{k}}\right)\;\frac{dt}{y_{i}}$$

By making the similar change of variables *s* = *t/y_i_*, (7) is given by

(8) $$\left(\prod_{k=1}^{i+1}\frac{y_{i}}{y_{k}}\right)\;\int_{1-z_{i+1}}^{1-z_{i+2}}{\left(1\;-\;s\right)}^{i}ds\;=\;\left(\prod_{k=1}^{i+1}z_{k}^{-1}\right)\;\left\{\frac{z_{i+1}^{i+1}\;-\;z_{i+2}^{i+1}}{i+1}\right\}$$

By continuing the similar computations for the probability of successful transmission during intervals [*y_i_* – *y_i_*_+2_, *y_i_* – *y_i_*_+3_], [*y_i_* – *y_i_*_+3_, *y_i_* – *y_i_*_+4_], . . ., we obtain the expression (3).

Let us consider a special case such that *y*_1_ ≥ *y*_2_ = *y*_3_ = . . . = *y_n_*. Let us denote the probability of Sensor 1 winning the frame by *p*_1_. From Proposition 1 it is given by

(9) $$p_{1}\;=\;1\;-\;z_{2}\;+\;\frac{1}{z_{2}^{n-1}}\frac{z_{2}^{n}}{n}\;=\;1\;-\left(\frac{n-1}{n}\right)\;z_{2}$$

where *z*_2_ = *y*_2_*/y*_1_. The probability of a Sensor other than Sensor 1 becoming the winner of frame is given by (1 – *p*_1_)/(*n* – 1) = *z*_2_*/n* = *y*_2_/(*ny*_1_) due to the symmetry among *n* – 1 sensors except Sensor 1. Thus these sensors gets the probability proportional to their score *y*_2_. Thus this can serve as a good approximation when *n* – 1 sensors have small scores which are similar to each other, and there is one sensor with a large score, thus the probability is approximately *y_i_/*(*ny*_1_). Note the analysis is based on the continuous approximation, in particular the collision has been ignored. The actual probability of successful transmission and Equation (3) will be compared in Section 5 via numerical methods.

### 4.2. Selective Forwarding

The second technique is called *selective forwarding*. The idea is that one can suppress unnecessary data transmission by exploiting the property of max function. Suppose at *j*th frame a sensor received a packet which needs to be forwarded to the parent of the sensor. Assume that the sensor has kept history of the maximum score among all the received data up to (*j* – 1)th frame. Suppose the score contained in the packet received at *j*th frame is less than the maximum score over all the packets which have been received and forwarded by the sensor prior to the *j*th frame. Since the sink needs to retrieve only the maximum score data, this packet will be eventually discarded at the sink. Rather than wasting resource to forward data to the sink, the sensor can silently drop the packet. This is called the selective forwarding. The max operation make such technique particularly simple, since it is easy to store the history of the maximum data.

**Figure 4.**
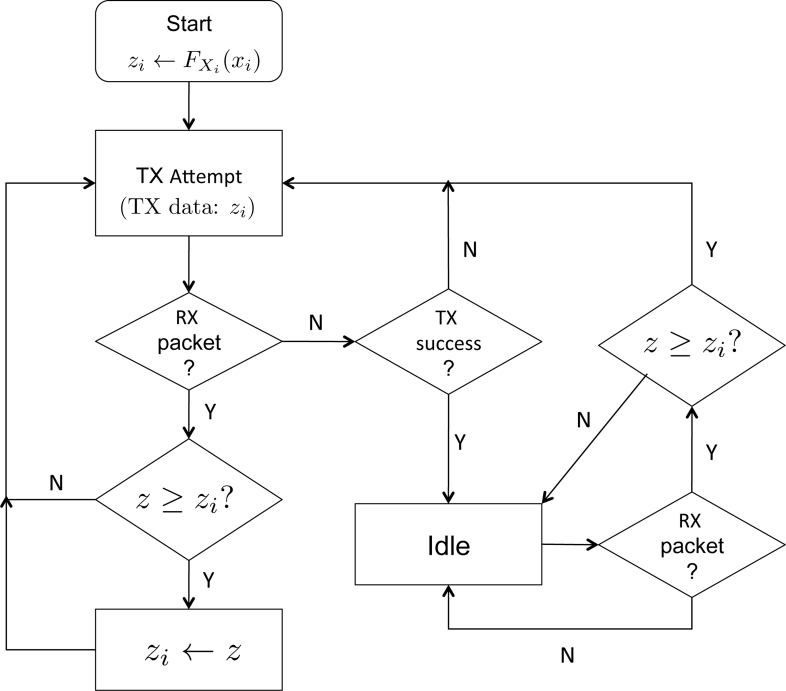
Procedure for selective forwarding. The data (score) of the received packet is denoted by *z*.

Let us denote the maximum among the scores which Node *i* has been successfully received for forwarding up to frame *t* ∈ ℤ_+_ by *z_i_*(*t*). We define *z_i_*(0) = *F*_*X*_i__ (*x_i_*). Suppose at frame *s* ∈ ℤ_+_, the node has received the data *z* requested for forwarding. In case where *z_i_*(*t*) > *z*, Node *i* will discard the data, and if *z_i_*(*t*) already has been transmitted, the sensor will go idle: otherwise the sensor will proceed to transmit *z_i_*(*t*). In case where *z_i_*(*t*) ≤ *z*, Node *i* updates *z_i_*(*s*) to *z*, and this data will be queued for transmission. A flowchart in Figure 4 illustrates the above procedure of selective forwarding. Note *z* in the flow chart denotes the received data (score). Also note that a sensor may receive data while attempting to transmit, and the procedure shown in Figure 4 captures such aspect combined with the selective forwarding.

**Figure 5.**
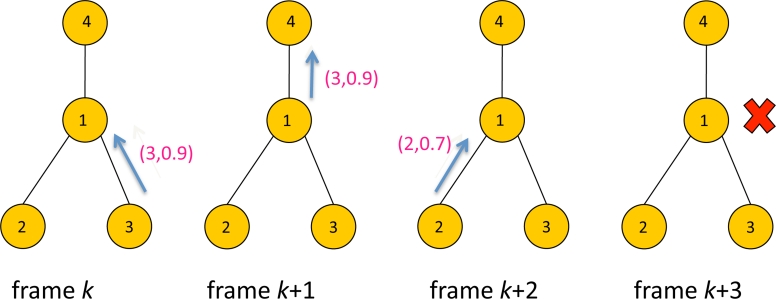
An example of selective forwarding.

Figure 5 illustrates an example of selective forwarding. There are four sensors with ID 1, 2, 3 and 4. The packet contains data with a format (*i, y_i_*) where *i* is sensor ID and *y_i_* is the score of the sensor. At frame *k*, Sensor 3 transmits the packet with data (3, 0.9) to Sensor 1. At frame *k* + 1, Sensor 1 forwards the data (3, 0.9) to its parent, Sensor 4. At this frame Sensor 1 updates *z*_1_(*k*) to the forwarded data 0.9, assuming *z*_1_(*k* – 1) < 0.9. At frame *k* + 2, Sensor 2 transmits data (2,0.7) to Sensor 1. However the score 0.7 is less than *z*_1_(*k*) = 0.9, Sensor 1 silently drops the packet and remains idle at frame *k* + 3. In this specific example only 3 packets have been transmitted using selective forwarding instead of 4 packets. Suppressing unnecessary packet transmissions will help reducing the contentions in upstream traffic to the sink, which will likely to reduce latency. This is demonstrated later in Section 5.

### 4.3. Exploiting Overhearing

Since the wireless channel is a shared medium, every transmission is in fact a broadcast. For a node with a close by transmitter, there is a chance of overhearing the transmitted packet even though the node is not the intended receiver. Overhearing is useful since such opportunity is obtained without contention, however a sensor can suppress transmission of unnecessary data. The scheme operates as follows. Similar to selective forwarding every sensor stores the maximum score up to current frame. Let us denote the maximum among all the score received/forwarded at Sensor *i* up to time *t* ∈ ℤ_+_ again by *z_i_*(*t*). If Sensor *i* overhears data *z* that is greater than *z_i_*(*t*) at frame *s*, the sensor can *update z_i_*(*s*) to *z*. Note such update increases *z_i_*(·), which increases the chance of discard unnecessary data using selective forwarding. Thus exploiting overhearing is a *complementary* scheme to selective forwarding.

Overhearing may not be always possible even though there is ongoing transmission by a neighboring sensor. The overhearing opportunity is affected by concurrent transmissions of other neighboring sensors, interference, *etc.* Given a set of transmitting sensors in the network, the set of nodes which can successfully overhear nearby transmission varies with the interference model used. The following is the condition under which overhearing is allowed in our node-exclusive interference model. Consider a transmitting sensor *i* and the set of neighboring sensors denoted by *N*(*i*). Any *v* ∈ *N*(*i*) can overhear *i*’s transmission if *v* is neither transmitting nor receiving at the same frame. The condition is from the limitation that in node-exclusive interference model, nodes cannot transmit and receive simultaneously. Note in other interference models such as two-hop interference model which is suitable for modeling IEEE 802.11 DCF (Distributed Coordination Function), one can find similar conditions for overhearing.

**Figure 6.**
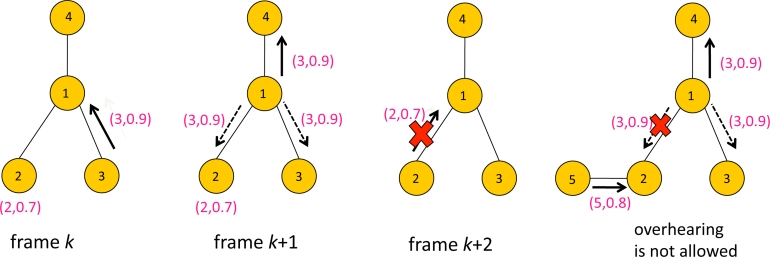
An example of overhearing.

Figure 6 illustrates an example of overhearing. There are four sensors with ID 1,2,3 and 4. The packet contains data with a format (*i*, *y_i_*) where *i* is sensor ID and *y_i_* is the score. At frame *k*, Sensors 2 and 3 contended for transmission to Sensor 1, and Sensor 3 won frame *k*. Thus Sensor 3 transmitted the packet with data (3, 0.9) to Sensor 1, while Sensor 2 waits for the next frame. At frame *k* +1, Sensor 1 forwards the data (3, 0.9) to its parent, Sensor 4. Sensors 2 and 3 are assumed to be idle, *i.e.*, neither transmitting or receiving at frame *k* + 1, which means they are eligible for overhearing the packet containing (3, 0.9) transmitted by Sensor 1. Having overheard data that is greater than the current data to transmit, Sensor 2 updates *z*_2_(*k* + 1) to 0.9. Since there is no need to forward (2,0.7) to Sensor 1, Sensor 2 remains idle at frame *k* + 2. Now suppose Sensor 2 had a child node. If Sensor 2 had been receiving data from its child sensor at frame *k* + 1, it would not have been able to overhear Sensor 1’s transmission, e.g., see the rightmost of Figure 6. In that case Sensor 2 might have transmitted at frame *k* + 2.

In general the graph describing the data gathering tree may not be the same as the node adjacency graph. Namely there can be a node adjacency graph *𝒢̂* such that the data gathering tree *𝒢* is a subgraph of *𝒢̂*. This implies that there may be further possible overhearing opportunity in the network other than that resulting from data gathering tree. For example, in Figure 6, Sensors 2 and 3 may be adjacent nodes in *𝒢̂*, although such relation is not specified in the forwarding graph *𝒢*. When the node adjacency graph is dense the network is a rich environment for overhearing. Note for dense graphs the network throughput may be severely limited by contention and interference. However overhearing compensates such limitation particularly for the applications retrieving the extreme data value, since unnecessary transmissions can be further suppressed by overhearing in denser networks, which in turn reduces subsequent contention for channel access.

The whole procedure which combines priority-based channel access, selective forwarding and overhearing is given in Algorithm 1. The SelectSlot() subroutine used in Algorithm 1 is provided in Algorithm 2.

### 4.4. Discussions

Recently distributed scheduling algorithms for wireless networks with contention based medium access has gained much interest. A series of work [14–21] has been proposed on assigning channel access priorities to nodes based on the individual loads, e.g., queue lengths. Note however, these studies have been carried out under different context: the emphasis has been on achieving a fraction of the *capacity region* of the network using only local information, e.g., queue lengths of contending neighbors. The capacity region is defined to be the union of the set of all input rates which can be supported by the network, *i.e.*, input loads under which the system can be stabilized.

Among these studies, [17] and [16] use timers with exponentially distributed timeouts as a means to assign priorities in channel access. If a node has data to transmit, it first sets a timer whose timeout is exponentially distributed independently of other nodes. The node waits until the timer expires, and begins transmission unless the channel is sensed to be busy. The rates of such exponential distribution are set according to certain function of the backlog of the nodes. Thus multiple neighboring nodes independently compete with exponentially distributed timers, and the one whose timer expires first wins the channel. Thus by controlling the rate of timer expiry, the priorities of channel access are implicitly assigned to nodes based on their individual loads.

A similar idea has been used for policy *V* [18] and Q-SCHED algorithm [20]. They also consider a two-phase contention model in which a node picks a minislot to transmit during contention phase. Nodes wait for a random number of minislots before transmission. The distribution of waiting time is a truncated version of geometric distribution which can be regarded as a discrete analogue of (truncated) exponential distribution. However in these schemes there is nonzero probability that a node does not attempt transmission [18,20]. In such schemes, since the nodes do not always participate in channel contention, the probability of collision will be relatively low. However one should be more aggressive in attempting transmissions in our problem, particularly for nodes with high scores. The reason is that, in the proposed scheme, selective forwarding and overhearing are used in addition to priority based channel access. This in turn increases the probability that high score nodes will be scheduled in the subsequent frames, *i.e.*, the effect is disseminated throughout the network. Once a transmission of high score packet is successful, it can suppress unnecessary traffic generated from the neighboring nodes. Also the system parameters *γ* and *ß* should be set in an aggressive way. Indeed we have heuristically found that setting *γ* = 3 and *ß* = 1 leads to better performance. Note when *γ* > 1 and as *ß* gets smaller, the sensors are more aggressive in transmission attempts. In summary, the synergistic effect among the components of the proposed scheme enables us to be more aggressive towards minimizing the latency.

Furthermore these works focus on achieving a long-term throughput that is sustainable by the network. However our problem pertains to retrieving information in a short period of time. The traffic is bursty in time and space, thus the regime of interest is quite different from such setup. Indeed we will demonstrate using simulation that those scheme may not be effective in reducing the latency of retrieving extreme statistics.

### 4.5. Extensions

In this subsection we discuss several extensions of the proposed framework. We first consider *k*-maximum data retrieval problem as follows. Let us denote *k*-th largest data from the scores *y*_1_, . . ., *y_n_* by *y*_[_*_k_*_]_. The *k*-maximum data retrieval problem is for the sink to collect *y*_[1]_, *y*_[2]_, . . ., *y*_[_*_k_*_]_. Similar overhearing and selective forwarding can be applied with a small change. The selective forwarding can be modified such that a node forwards only the data larger than the *k*-th largest data that it has received/forwarded. Note every sensor needs to maintain only *k* sets of variables to keep track of the history of *k* largest data since max is a divisible function. Similar modification can be made for the overhearing technique.

Another extension is a scenario where the sensors generate data samples periodically. Assume the sensors generate data samples at every unit time. When the signal for data retrieval is broadcast to the network, the sensors report the most recent data samples, say the last *b* samples, to the sink. The time instants of data sample generations are called *timestamps*. Thus the data samples are periodically generated and stored over a sliding time window of the length of *b* timestamps where the timestamps are one unit time apart. The goal of sink is to retrieve *b* maximum scores, one for each timestamps. Specifically suppose the timestamps for the maximum data retrieval are denoted by *t*_1_, *t*_2_, . . ., *t_b_*. Denote the score at sensor *i* for timestamp *t_j_* by *y_i_*(*t_j_*). The objective for the sink is to collect y*(*j*) = max*_i_*_∈_*_I_* [*y_i_*(*t_j_*)] for *j* = 1, 2, . . ., *b*. The latency is measured as the time when all of *b* maximum scores y*(*j*), *j* = 1, 2, . . ., *b* are retrieved at the sink. We assume a packet can contain only one data sample, and only one packet can be transmitted per frame. The timestamp is assumed to be included in a packet. In this application the sensors can apply rules for selective forwarding and overhearing separately to packets with different timestamps. A set of packets with the same timestamp can be treated as an individual *flow*, thus the proposed scheme can be applied separately to each flow. However the latency is affected by the parameter *b*. Since initially *b* packets need to be transmitted by every node, when *b* is large the network may undergo congestion for a long period of time. We later study the impact of *b* on the performance using simulations.

## 5. Simulation

### 5.1. Latency Distribution

By simulation we demonstrate the efficacy of the proposed scheme. We assume a complete binary tree as the data gathering tree. The number of nodes considered have been *n* = 2^7^ – 1 = 127 and *n* = 2^9^ – 1 = 511. The number of minislots were varied from either *m* = 10 or *m* = 30. We compare the performance of the proposed scheme with a baseline scheme defined as follows. The baseline scheme uses UNIFORM scheme for the medium access, and uses a simple in-network aggregation of combining available data without selective forwarding or overhearing. The latency has been measured in terms of the number of frames it takes for the extreme data arrives at the sink. Recall the latency is denoted by a RV *T*, and for a given deadline *d* we would like to maximize ℙ(*T = d*). Thus we examine the CDF of the latency for comparison.

**Figure 7.**
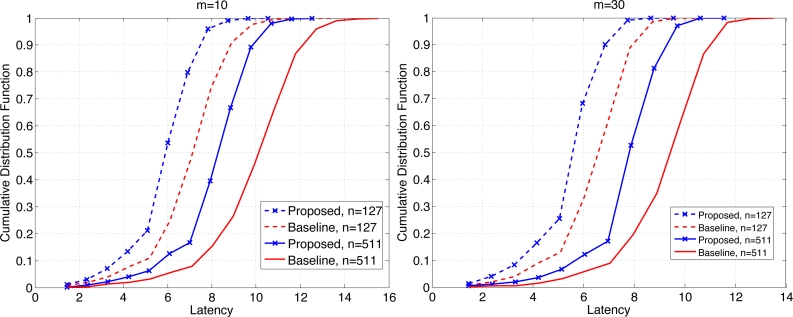
Comparison of CDF of latency for *m* = 10 and 30 when *b* = 1.

Figure 7 shows the CDF of the latency when *b* = 1 for *n* = 127 and 511, each for *m* = 10 (left) and 30 (right). We see that the proposed scheme clearly outperforms the baseline scheme. For example when the probability of retrieval of the maximum data is given by 0.8, the proposed scheme achieves the latency that is roughly 19%–20% (resp. 15%–17%) lower than the baseline scheme when *m* = 10 (resp. *m* = 30). The overall latency is reduced for larger *m* since the contention window has become wider. The relative performance of the proposed scheme is slightly better for smaller *m*, which suggests that as the level of contention become higher, the proposed scheme becomes more effective.

**Figure 8.**
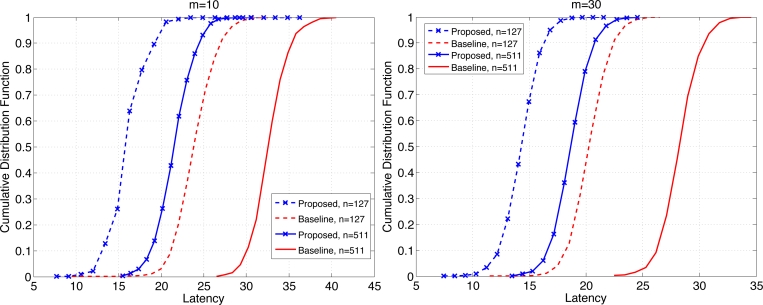
Comparison of CDF of latency for *m* = 10 and 30 when *b* = 3.

Figure 8 shows the CDF of the latency when *b* = 3 for *n* = 127 and 511, each for *m* = 10 (left) and 30 (right). The reduction in latency at the probability of retrieval of 0.8 is 32–33% (resp. 30–32%) when *m* = 10 (resp. *m* = 30). Clearly the gains have increased compared to the case where *b* = 1. With larger *b* the network undergoes higher level of contention and temporary congestion, thus the gain in latency reduction due to the proposed scheme increases under high load on the network. The mean latency has been compared in Table 1. The relative reduction in mean latency by the proposed scheme ranges 15.1–18.0% when *b* = 1, and 31.1–34.3% when *b* = 3.

### 5.2. Comparisons of Random Access Schemes

Note that there are many ways to assign priorities of channel access to nodes. In this subsection we compare several schemes for priority assignment based on previous work on multi-hop networks [18,20]. These works also adopt a two-phase time slotted model for channel access. The contention window is similarly defined as consisting of a fixed number of minislots. The key difference is the algorithms for choosing a minislot for transmission. We apply these algorithms to our problem and compare the performance with the proposed scheme. We first need the following definition. Let us denote the set of the child nodes of Sensor *i* by *𝒞*(*i*) and the parent node by *𝒫*(*i*) in the aggregation tree. Note the nodes in *𝒞*(*i*) and *𝒫*(*i*) are the interferers to Sensor *i*. Let us define

(10) $$q_{i}\;=\;\frac{y_{i}}{y_{i}\;+\;\text{max}\left(y_{𝒫(i)},\;\sum_{k\in 𝒞(i)}y_{k}\right)}$$

Roughly speaking *q_i_* can be viewed as a ‘normalized’ score of Sensor *i* with respect to its interferers.

The first algorithm we consider is policy *V* proposed in [18]. In policy *V* a node attempt a transmission at every minislot with the following probability:

(11) $$\left(\frac{\sqrt{m}\;-\;1}{2m}\right)\;\cdot \;q_{i}$$

The second algorithm is Q-SCHED [20]. In the algorithm a discrete RV *M* is defined which takes a value from {1, 2, . . ., *m* + 1} and has the following distribution: for *k* = 1 . . ., *m*,

(12) $$\mathbb{P}(M=k)\;=\;\text{exp}\left(-\frac{g(m)q_{i}}{m}(k\;-\;1)\right)\;-\;\text{exp}\;\left(-\frac{g(m)q_{i}}{m}k\right)$$

where *g*(*m*) = log(2*m*)/2. ℙ(*M* = *k*) is the probability that the minislot *k* is selected. Also $\mathbb{P}(M\;=m+1)\;=\;1\;-\;\sum_{k=1}^{m}\mathbb{P}(M\;=\;k)$ is defined to be the probability that the node will not attempt transmission in the frame. Note the algorithms above are modified to fit our model: in the original definition of *q_i_*, instead of scores, a ratio of queue lengths normalized by link capacity is used. The analysis of [18,20] shows that with the above algorithm, the probability of Sensor *i* becoming the winner is guaranteed up to an amount that is proportional to *q_i_*. Specifically the probability is at least *q_i_*(0.5 – *f* (*m*)) where *f* (*m*) → 0 as *m* → ∞. Roughly speaking, the priority of channel access is assigned to Sensor *i* such that the channel access is guaranteed with a probability close to 0.5*q_i_*.

**Figure 9.**
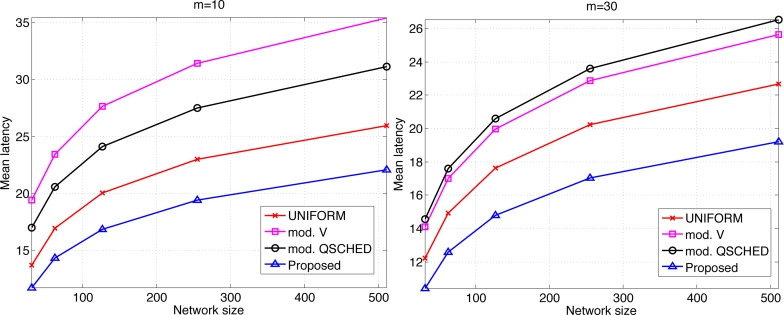
Comparison of the mean latency for different random access schemes.

**Figure 10.**
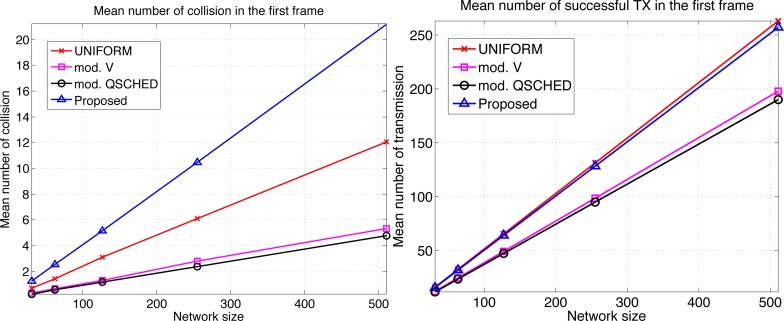
Mean number of collision and successful transmissions in the first frame when *m* = 30 and *b* = 3.

**Figure 11.**
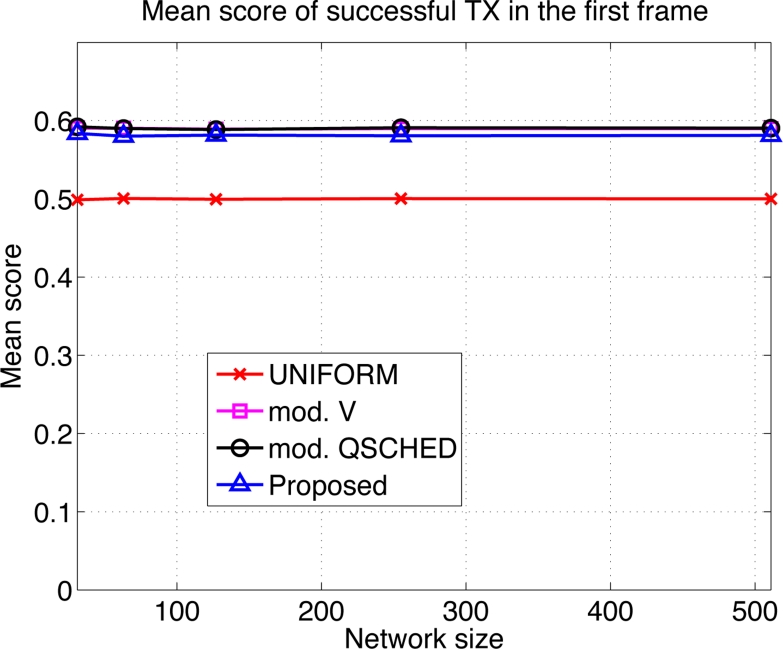
Mean score of successful transmissions in the first frame when *m* = 20 and *b* = 3.

We compare the performances of different algorithms for channel access. Figure 9 shows the mean latency associated with the channel access schemes. The selective forwarding and overhearing are used with varying medium access schemes. ‘Mod. *V*’ (resp ‘Mod. Q-SCHED’) represents the modified policy *V* (resp. Q-SCHED algorithm). We observe that the proposed scheme incurs the lowest mean latency both for *m* = 10 and 30. Somewhat surprisingly, UNIFORM scheme performs better than modified policy *V* and Q-SCHED. The result can be explained as follows. Figure 10 shows the statistics on the number of collision and the amount of traffic generated at the first frame. The first frame is defined to be the frame at which the data gathering is initiated. Note the generated traffic is potentially the heaviest at the first frame. On the left of Figure 10 is the mean number of collisions in the first frame. We see that the number of collision of the proposed scheme is higher than UNIFORM scheme, while Q-SCHED and policy *V* have considerably lower number of collisions. However seemingly high number of collisions for the proposed scheme is in fact relatively small compared to the number of successful transmissions in the first frame which is shown on the right of Figure 10. We observe that the number of successful transmissions in the first frame for policy *V* and Q-SCHED are lower than the proposed and UNIFORM scheme. The reason is that, under policy *V* and Q-SCHED algorithm, there is nonzero probability that a node is ‘idle’, *i.e.*, it does not participate in channel contention. While in the proposed and UNIFORM scheme, every node attempts transmission at every frame. This also accounts for the lower number of collisions for policy *V* and Q-SCHED algorithm. Figure 11 shows the mean score of the successful transmitted packet in the first frame. Since the scores of sensors in the first frame are *n* i.i.d. uniform RVs on [0,1] by definition, we see that the mean score for UNIFORM scheme is approximately 0.5. We see that the modified policy *V* and Q-SCHED has a mean score that is only slightly higher than the proposed scheme, which is possible however, by reducing the number of successful transmissions in the first frame. Figure 11 shows that the proposed scheme finds compromise on these aspects: it achieves higher mean score than UNIFORM scheme without excessively reducing the number of successful transmissions in the first frame. Although not shown here, we observed that the number of collisions subsides faster in subsequent frames for the proposed scheme, compared to other schemes. This is a synergistic effect with selective forwarding and overhearing, since successful transmissions of high-score data contribute to suppressing traffic and reducing contention by these techniques, which eventually leads to decreased latency.

Here we make an important observation. When one intends to further prioritize high-score sensors, it is likely to lower the number of successful transmissions. Reducing the number of successful transmissions, especially by increasing the number of ‘idling’ nodes, seems to be more harmful to latency reduction as we see that UNIFORM outperforms policy *V* and Q-SCHED in Figure 9. Thus it is crucial that one should aggressively prioritize high score nodes, however should not excessively lower the number of successful transmissions. The proposed scheme is observed to exhibit such property.

### 5.3. Analytical Estimate

**Figure 12.**
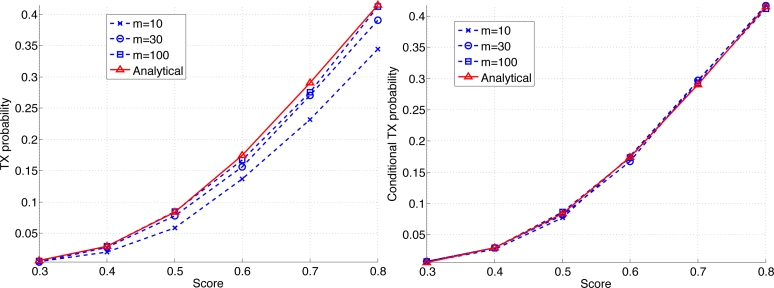
Probability of a successful transmission as a function of the scores.

In this subsection we study the effect of a finite *m* on the analytical estimate Equation (3). We consider a set of nodes with *n* = 6 having scores (0.3,0.4,0.5,0.6,0.7,0.8). We assume every node interferes with every other node. The probability of winning a frame under the proposed medium access scheme is numerically computed. On the left of Figure 12 is a plot of the probability of winning a frame as a function of the score with varying *m*. For small *m*, there is a gap between the probability of winning and the analytical estimate. This is due to the fact that the analysis assumes *m* → ∞ and does not account for collisions. However the gap appears to be small for a substantially small value of *m*, e.g., *m* = 10, considering the relatively large number of contending users, *i.e.*, *n* = 6. For larger value of *m* the estimate becomes fairly accurate.

Let us consider the probability of winning a frame *conditional on* that no collision has occurred. Heuristically one could expect that Equation (3) becomes a better estimate, since by definition the collisions are already accounted for when the conditional probability is computed. On the right of Figure 12 is a plot of the probability of successful transmissions conditional on that no collision has occurred. Indeed the figure seems to support the argument: Equation (3) is virtually identical to the actual conditional distributions for all the simulated values of *m*. Let us denote the probability of Sensor *i* winning a frame for finite *m* by *p_i_*. We conclude that Equation (3) can be used as an approximation to *p_i_/*Σ*p_i_*. In summary the analytical estimate Equation (3) is a reasonably good approximation even for small *m*, and is an excellent approximation for the conditional distribution, *i.e.*, the *relative* probability of a successful transmission among sensors.

## 6. Conclusions

In the work we have proposed the schemes to reduce latency of retrieving the extreme data from sensor networks. We have proposed three techniques: priority based medium access, selective forwarding and exploiting overhearing. By combining these techniques one can effectively explore the trade-off between prioritizing high score data and moderating contention. The proposed scheme is able to suppress unnecessary traffic and reduce contention, thus can be more aggressive in assigning high priority to high score data. The simulation results demonstrate that significant gains in the reduction of latency can be achieved by the proposed scheme.

## Figures and Tables

**Table 1.** Comparison of mean latency.

	*m* = 10		*m* = 30	
	Baseline	Proposed	Baseline	Proposed
*n* = 127, *b* = 1	7.41	6.25	6.92	5.89
*n* = 511, *b* = 1	10.57	8.67	9.70	8.24
*n* = 127, *b* = 3	24.32	16.80	21.24	14.81
*n* = 511, *b* = 3	33.20	21.82	28.84	19.15

**Algorithm 1:** The proposed scheme.

*z_i_* ← *F*_*X_i_*_ (*x_i_*), FrameCount ← 1, *c* ← 0
NeedToTX←TRUE
TXSlot←SelectSlot() // Minislot selection
**loop** // Begin infinite loop
SlotCount← 1
**while** SlotCount≤ *m***do** // Begin SlotCount loop
**if** (detect that medium is busy) **then**
**if** (this node is the receiver) **then**
emsp; [Receive the packet]
*z* ← received data
**if***z* ≥ *z_i_***then** // Selective forwarding
NeedToTX← TRUE
*z_i_* ← *z*
TXSlot← SelectSlot() // Minislot selection
**end if**
**else**
**if** (overhearing is possible) **then** // Overhearing
[Overhear the transmitted packet]
*z* ← overheard data
**if***z* ≥ *z_i_***then**
NeedToTX← FALSE
*z_i_* ← *z*
**end if**
**end if**
**end if**
SlotCount← *m* // Exit from SlotCount loop
**else**
**if** (NeedToTX=TRUE) & (SlotCount=TXSlot) **then**
[Transmit packet with data *z_i_*]
**if** (Collision occurred) **then**
*c* ← *c* + 1
**else**
NeedToTX← FALSE // Successful transmission
*c* ← 0
**end if**
SlotCount← *m* // Exit from SlotCount loop
**end if**
**end if**
SlotCount←SlotCount+1
[Wait until the next minislot starts]
**end while** // End SlotCount loop
FrameCount←FrameCount+1
[Wait until the next frame starts]
**end loop** // End infinite loop

**Algorithm 2:** SelectSlot() subroutine.

*w* ← min {⌈2*^c^* · *m* · (*y_i_*)^γ^ ⌉ + *ß*, *m*}
**return** (a randomly selected integer from *m* – *w* + 1, . . ., *m*)
